# Comparative efficacy and safety of urate-lowering therapy for the treatment of hyperuricemia: a systematic review and network meta-analysis

**DOI:** 10.1038/srep33082

**Published:** 2016-09-08

**Authors:** Shu Li, Hongxi Yang, Yanan Guo, Fengjiang Wei, Xilin Yang, Daiqing Li, Mingzhen Li, Weili Xu, Weidong Li, Li Sun, Ying Gao, Yaogang Wang

**Affiliations:** 1 Department of Health Service & Care Management, School of Public Health, Tianjin Medical University, Tianjin, 300070, China; 2 Metabolic Disease Hospital & Tianjin Institute of Endocrinology, Tianjin Medical University, Tianjin, 300070, China; 3 Research Center of Basic Medical Sciences, Tianjin Medical University, Tianjin, 300070, China; 4 Department of Epidemiology and Biostatistics, School of Public Health, Tianjin Medical University, Tianjin, 300070, China; 5 Aging Research Center, Department of Neurobiology, Care Sciences and Society, Karolinska Institutet and Stockholm University, Stockholm, Sweden

## Abstract

The prevalence of hyperuricemia and gout has been increasing, but the comparative effectiveness and safety of different treatments remain uncertain. We aimed to compare the effectiveness and safety of different treatments for hyperuricemia using network meta-analysis methodology. We systematically reviewed fifteen randomized controlled trials (involving 7,246 patients through January 2016) that compared the effects of different urate-lowering drugs (allopurinol, benzbromarone, febuxostat, pegloticase and probenecid) on hyperuricemia. Drug efficacy and safety, as outcomes, were measured by whether the target level of serum urate acid was achieved and whether any adverse events occurred, respectively. We derived pooled effect sizes expressed as odds ratios (ORs) and 95% confidence intervals (CIs). The efficacy and safety of the drugs were ranked by cumulative ranking probabilities. Our findings show that febuxostat, benzbromarone, probenecid, pegloticase, and allopurinol were all highly effective at reducing the risk of hyperuricemia compared to placebo. Febuxostat had the best efficacy and safety compared to the other drugs. Furthermore, febuxostat 120 mg QD was more effective at achieving urate-lowering targets (OR: 0.17, 95% CI: 0.12–0.24) and safer (OR: 0.72, 95% CI: 0.56–0.91) than allopurinol.

Hyperuricemia (HUA), defined as a serum urate concentration exceeding the limit of solubility (approximately 6.8 mg/dl), is considered a common biochemical abnormality that reflects supersaturation of the extracellular fluid with urate^1^. The Global Burden of Disease (GBD) 2010 Study reported that the global prevalence of gout was 0.08%^2^. Recent epidemiological studies have shown evidence that hyperuricemia and gout cases have continued to grow for decades^3^. In view of the rapid economic development and the magnitude of populations, the prevalence rate has increased noticeably in developing countries, such as China^4^,^5^. There were 15.3 million who were diagnosed with chronic gout in major countries in 2013, and the number with gout is projected to be 17.7 million in 2021^6^. Hyperuricemia results either from the overproduction of uric acid (10%) or the under-excretion of urate (90%)^7^, leading to the deposition of monosodium urate crystals in and around the joints^8^,^9^. Thus, elevated serum urate acid (sUA) levels increase the risk of gout and various comorbidities^10^,^11^,^12^,^13^,^14^,^15^.

Urate-lowering therapy (ULT) has been widely used to control hyperuricemia and prevent gout. The 2012 American College of Rheumatology (ACR) recommended that the target of ULT was to achieve a sUA level <6 mg/dl in all gout patients or a sUA <5 mg/dl for gout patients with tophi^16^. Anti-hyperuricemia drugs can be classified into three groups based on their pharmacologic mechanism. Uricosuric drugs are inhibitors of uric acid synthesis and the enzyme uricase. Among uricosuric agents, probenecid is commonly used^16^, whereas benzbromarone has been withdrawn in most European countries since 2003 due to serious hepatotoxicity^17^. Despite its adverse effects, benzbromarone is still applied commonly in clinics in several countries in Asia, including China^18^. Allopurinol, febuxostat and in particular Xanthine oxidase inhibitors (XOIs) are recommended as first-line drugs^16^. However, allopurinol has been reported to be associated with severe cutaneous adverse reactions^19^. In fact, humans lack urate oxidase, an enzyme that catalyses the oxidation of uric acid to allantoin, consequently resulting in hyperuricemia if accumulated in the blood^20^. Pegloticase, a recombinant polyethylene glycol conjugate of uricase (PEG-uricase), has been approved for the treatment of refractory chronic gout in the US and European Union^21^.

In 2014, a panel of 78 international rheumatologists raised ten key clinical questions pertinent to the diagnosis and management of gout, and one of these questions was how to determine the efficacy, cost-efficacy and safety of ULT (allopurinol, benzbromarone, febuxostat, peg-uricase and probenecid) in the treatment of gout^22^. Two previous pairwise meta-analyses analysed available individual studies and suggested that febuxostat may be associated with better urate lowering efficacy than allopurinol^23^,^24^. However, traditional meta-analyses can only draw interactions between comparisons among treatments with valid head-to-head trials^25^,^26^. Currently, several network-based approaches have been applied to potential drug discovery and biological information mining, e.g., drug–target interaction identification^27^,^28^, drug similarity calculation^29^ and genome-disease function inference^30^. As an extension of a pairwise meta-analysis, a network meta-analysis provides a method for assessing the relative effectiveness of two treatments when they have no direct comparison in randomized trials^31^. Herein, we performed a network meta-analysis to evaluate the comparative efficacy and safety of five urate-lowering drugs, focusing on their ability to achieve target serum urate acid levels and the risk of adverse events.

## Methods

### Search strategies and selection criteria

This systematic review was conducted in accordance with the Preferred Reporting Items for Systematic Reviews and Meta-analyses (PRISMA) extension statement for network meta-analyses of health care intervention studies^32^. The PubMed, Medline, Embase, Cochrane Library databases and ClinicalTrials.gov were searched from inception to Jan 16, 2016. Following the PICOS (Participants, Interventions, Comparisons, Outcomes and Study design) principle^33^, the key search terms included (P) hyperuricaemia, hyperuricemia, gout, (I) urate-lowering therapy, uric acid, urate, (C/O) allopurinol, benzbromarone, febuxostat, pegloticase, probenecid, and (S) random*, and randomized controlled trial.

Studies meeting the following criteria were included: (a) Patients: adults (age >18 years old) with hyperuricemia with or without chronic gout; (b) Intervention: established ULT with at least one of five agents (allopurinol, benzbromarone, febuxostat, pegloticase or probenecid); (c) Comparator: placebo or another agent of the five mentioned above; (d) the outcome of efficacy was defined as a failure to achieve the sUA treatment target level, i.e., ≤6 mg/dl (or 360 μmol/l) with ULT, and the outcome of safety was defined as any adverse events during the period of the trial, including abnormal liver function, renal impairment, hyperlipidaemia, diarrhoea, gastrointestinal disorders, joint-related signs and symptoms; and (e) Study design: randomized controlled trial (RCT). The exclusion criteria were as follows: (a) trials comparing different doses of the same medication only; (b) studies without a designated intervention/comparator arm; (c) animal experiments; and (d) studies reported in a language other than English.

**Figure 1:**
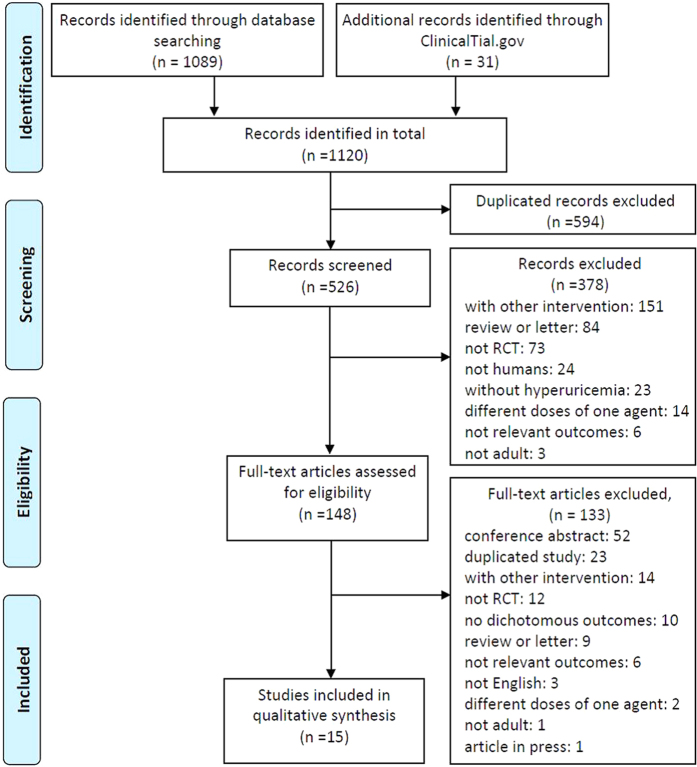
Flow diagram of literature search and selection

Three researchers (S.L., H.X.Y. and Y.N.G.) independently screened all records according to the inclusion and exclusion criteria. Any inconsistencies were resolved by discussion among the three authors. Finally, we identified fifteen qualified RCTs that were included in the current analysis^1^,^34^,^35^,^36^,^37^,^38^,^39^,^40^,^41^,^42^,^43^,^44^,^45^,^46^,^47^. The complete process and the exclusion reasons are shown in Fig. 1.

### Data extraction and quality assessment

Two investigators (S.L., H.Y.) reviewed the full text of the eligible studies and extracted information into an electronic database. The information included study design, patient characteristics, inclusion/exclusion criteria, treatment protocols, and outcomes (the number of patients with/without successful treatment and occurrence of adverse events, which were available as binomial counts (successes/total)). The information was double checked by referring to the original articles when an inconsistency existed.

The quality of the included studies was reviewed and assessed by two investigators (S.L., Y.G.) independently using the Cochrane Collaboration’s tool for assessing the risk of bias^48^. A risk of bias graph displays the grade of bias as high risk, unclear risk and low risk (see Supplementary Figure 1A,B). Five studies were considered to have a high risk of bias due to the lack of implementation of blinding^37^,^41^,^42^,^43^,^47^.

### Data synthesis and analysis

We calculated the odds ratios (ORs) and 95% confidence intervals (95% CIs) of the drugs for their failure to achieve the sUA treatment target level and related adverse events. Head-to-head meta-analysis was used to generate direct evidence (from studies directly comparing A to B). The pairwise meta-analysis with random-effect models was performed, and statistical heterogeneity was estimated using I^2^ statistics, which describe the percentage of variability across studies caused by heterogeneity rather than chance^49^.

In addition to direct evidence, we also drew inferences between two intervention arms, such as A versus B, from indirect evidence (from combining studies through an intermediate comparator C, e.g., A *vs.* C and B *vs.* C studies)^50^,^51^. With the use of the adjusted indirect comparison method and inverse variance method, the effect estimates between treatments without direct comparisons and the combined results of direct and indirect evidence were obtained, respectively. Thus, even if there are no known comparisons for the investigated drug, a network meta-analysis still can estimate the potential effect of this drug based on existing head-to-head trials. As a result, a synthesized effect size and mean rank could be estimated for all the interventions.

When conducting a network meta-analysis, three assumptions need to be met, including homogeneity, transitivity, and consistency. The treatment effects together with their predictive intervals (PrIs) are examined to illustrate the magnitude of heterogeneity. A predictive interval plot is drawn to make comparisons between the 95% CIs and the 95% PrIs. The transitivity (or named similarity) assumption refers to the balance between the relative treatment effects and covariates across trials that are comparing different sets of interventions^52^. The inconsistency accounts for disagreements between direct and indirect evidence^53^. It is generally recommended to evaluate the consistency assumption using both global and local approaches. To assess the assumption of consistency in the entire network, we inferred the presence of inconsistency from any source in the entire network based on a Chi-square test. To evaluate the presence of inconsistency locally, we used the loop-specific approach to evaluate the inconsistency factor (IF, the difference between the direct and indirect estimate for one of the comparisons in a particular loop). We identified inconsistency as yielding a lower 95% CI limit that does not reach the zero line.

To rank the treatments based on efficacy and safety, we calculated the probabilities of the surface under the cumulative ranking curve (SUCRA). SUCRAs can illustrate the outcome percentages of every treatment relative to an ideal treatment, which always ranks first without uncertainty. Thus, the greater the SUCRA score, the more effective or safer the drug.

We performed the network meta-analysis using a frequentist model. Stata version 13 was used to make calculations. The metan and network commands were used for the pairwise and network meta-analyses, respectively. In the network meta-analysis, zero cells were corrected with the command “network setup” in Stata.

## Results

### Characteristics of eligible studies

Fifteen studies involving 7,246 adult trial subjects were included in the network meta-analysis. The characteristics of the included studies are summarized in Table 1. The earliest study was conducted in 1999, whereas the latest one was in 2016. The duration of the trials ranged from 4 to 52 weeks. Seven trials made a comparison between allopurinol and febuxostat^1^,^35^,^36^,^37^,^38^,^46^,^47^, two trials between allopurinol and benzbromarone^41^,^43^, one trial between benzbromarone and probenecid^42^, three trials between febuxostat and placebo^34^,^39^,^40^, and one trial between pegloticase and placebo^45^. A three-arm trial compared allopurinol, febuxostat and placebo^44^. The dosage of febuxostat among the trials ranged from 20 mg/day to 240 mg/day. In general, all of the trial patients had an average age of 30 or more years, and males accounted for more than 80% of the subjects in the included trials. At baseline, these trial subjects had sUA concentrations >8.0 mg/dl.

**Figure 2:**
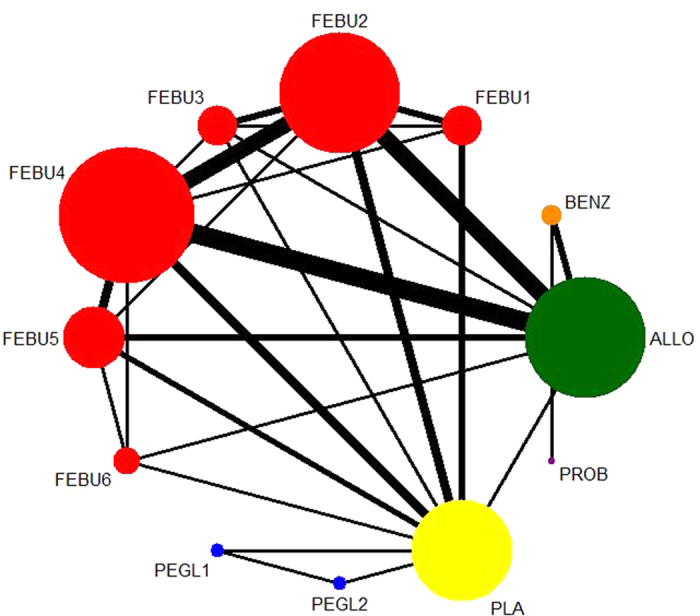
Network meta-analysis for indirect treatment comparisons. The network geometry is composed of nodes and edges. The size of nodes and the thickness of edges were weighted by the sample size and number of trials, respectively. A lack of lines indicates that there were no head-to-head trials between two treatments. ALLO = allopurinol, FEBU1 = febuxostat 20 mg/day, FEBU2 = febuxostat 40 mg/day, FEBU3 = febuxostat 60 mg/day, FEBU4 = febuxostat 80 mg/day, FEBU5 = febuxostat 120 mg/day, FEBU6 = febuxostat 240 mg/day, BENZ = benzbromarone, PROB = probenecid, PEGL1 = pegloticase 8 mg every 2 weeks, PEGL2 = pegloticase 8 mg every 4 weeks, PLA = placebo.

A network graphical structure displays the available direct comparisons of the network of trials organized from the fourteen RCTs (Fig. 2). Comparisons with febuxostat (20/40/60/80/120/240 mg once daily) or pegloticase (8 mg every two/four weeks) were classified by dosage.

### Direct treatment comparisons

#### Pairwise meta-analysis

The pairwise meta-analysis showed that allopurinol, febuxostat 20/40/60/80/120/240 mg QD (20/40/60/80/120/240 mg once daily), and pegloticase 8 mg 2 W/4 W (8 mg every two/four weeks) were all highly effective at achieving the sUA treatment target compared to placebo (Table 2). Febuxostat was more likely to achieve the sUA treatment target than allopurinol (OR of allopurinol *vs.* febuxostat 40 mg QD: 1.29, 95% CI: 1.05–1.59; OR of allopurinol *vs.* febuxostat 80 mg QD: 3.62, 95% CI: 2.69–4.89; OR of allopurinol *vs.* febuxostat 120 mg QD: 6.34, 95% CI: 4.79–8.40; and OR of allopurinol *vs.* febuxostat 240 mg QD: 18.31, 95% CI: 9.17–36.58). Febuxostat 40/60/80 mg QD showed better efficacy than febuxostat 20 mg QD (OR of febuxostat 20 *vs.* 40 mg QD: 7.39, 95% CI: 3.29–16.63; OR of febuxostat 20 *vs.* 60 mg QD: 5.79, 95% CI: 1.99–16.63; and OR of febuxostat 20 *vs.* 80 mg QD: 8.28, 95% CI: 2.73–25.15). Febuxostat 80/120 mg QD showed better efficacy than febuxostat 40 mg QD (OR of febuxostat 40 *vs.* 80 mg QD: 2.28, 95% CI: 1.92–2.70; and OR of febuxostat 40 *vs.* 120 mg QD: 12.63, 95% CI: 2.60–61.38). Febuxostat 120/240 mg QD showed better efficacy than febuxostat 80 mg QD (OR of febuxostat 80 *vs.* 120 mg QD: 1.48, 95% CI: 1.05–2.08; and OR of febuxostat 80 *vs.* 240 mg QD: 4.44, 95% CI: 2.20–8.96). Febuxostat 240 mg QD showed better efficacy than febuxostat 120 mg QD (OR of febuxostat 120 *vs.* 240 mg QD: 3.11, 95% CI: 1.53–6.32). Regarding safety, allopurinol was more likely to cause adverse events than febuxostat 120 mg QD (OR of allopurinol *vs.* febuxostat 120 mg QD: 1.56, 95% CI: 1.17–2.08). There were no statistically significant differences among the other treatments identified by the direct comparisons.

#### Heterogeneity

Substantial heterogeneity was observed when comparing benzbromarone (I^2^ = 73.7%) or febuxostat 80 mg QD (I^2^ = 69.3%) with allopurinol for efficacy. Nevertheless, there was no evidence showing heterogeneity in the other pooled results of the direct comparisons for either efficacy or safety.

### Network estimation and ranking

#### Network treatment comparisons

**Figure 3:**
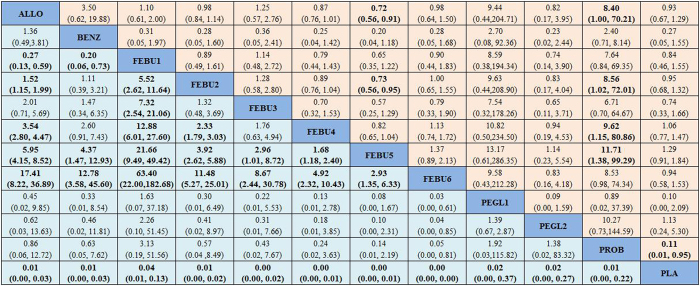
Odds ratios (ORs) with corresponding 95% confidence intervals (95% CIs) for efficacy and safety of drugs according to network estimates. Treatments are reported in efficacy-ranking order. Data in light blue represent efficacy, and the column treatment is compared with the row treatment. For efficacy, OR less than 1 favours the treatment in the column. Data in pink are results on safety, where the row treatment is compared with the column treatment. For safety, OR less than 1 favours the treatment in the row. The results with significant differences are bold. ALLO = allopurinol, FEBU1 = febuxostat 20 mg/day, FEBU2 = febuxostat 40 mg/day, FEBU3 = febuxostat 60 mg/day, FEBU4 = febuxostat 80 mg/day, FEBU5 = febuxostat 120 mg/day, FEBU6 = febuxostat 240 mg/day, BENZ = benzbromarone, PROB = probenecid, PEGL1 = pegloticase 8 mg every 2 weeks, PEGL2 = pegloticase 8 mg every 4 weeks, PLA = placebo.

Pooled ORs and 95% CIs for the efficacy and safety of the different interventions from the network meta-analysis are shown in Fig. 3. Febuxostat, benzbromarone, probenecid, pegloticase and allopurinol were all highly effective in comparison to placebo at achieving the treatment target. Febuxostat was mostly more effective than allopurinol at achieving the treatment target of hyperuricemia (OR of allopurinol *vs.* febuxostat 40 mg QD: 1.52, 95% CI: 1.15–1.99; OR of allopurinol *vs.* febuxostat 80 mg QD: 3.54, 95% CI: 2.80–4.47; OR of allopurinol *vs.* febuxostat 120 mg QD: 5.95, 95% CI: 4.15–8.52; and OR of allopurinol *vs.* febuxostat 240 mg QD: 17.41, 95% CI: 8.22–36.89) except febuxostat 20 mg QD (OR of allopurinol *vs.* febuxostat 20 mg QD: 0.27, 95% CI: 0.13–0.59). Benzbromarone was found to have better efficacy than febuxostat 20 mg QD (OR of benzbromarone *vs.* febuxostat 20 mg QD: 0.20, 95% CI: 0.06–0.73) but was worse than febuxostat 120/240 mg QD (OR of benzbromarone *vs.* febuxostat 120 mg QD: 4.37, 95% CI: 1.47–12.93; and OR of benzbromarone *vs.* febuxostat 240 mg QD: 12.78, 95% CI: 3.58–45.60). The urate-lowering efficiency of febuxostat improved with increasing dosages. Regarding safety, the incidence of adverse events was less using febuxostat 120 mg QD than allopurinol or febuxostat 40 mg QD (OR of febuxostat 120 mg QD *vs.* allopurinol: 0.72, 95% CI: 0.56–0.91; and OR of febuxostat 120 mg QD *vs.* febuxostat 40 mg QD: 0.73, 95% CI: 0.56–0.95). Probenecid had more occurrences of adverse events than allopurinol, febuxostat 40/120/240 mg QD or placebo (OR of probenecid *vs.* allopurinol: 8.40, 95% CI: 1.00–70.21; OR of probenecid *vs.* febuxostat 40 mg QD: 8.56, 95% CI: 1.02–72.01; OR of probenecid *vs.* febuxostat 80 mg QD: 9.62, 95% CI: 1.15–80.86; OR of probenecid *vs.* febuxostat 120 mg QD: 11.71, 95% CI: 1.38–99.29; and OR of placebo *vs.* probenecid: 0.11, 95% CI: 0.01–0.95). Other comparison results were not significant statistically.

#### Heterogeneity and inconsistency

The 95% PrI and 95% CI of each pairwise comparison are displayed in Supplementary Figure 2. There was no clear evidence suggesting inconsistency between the direct and indirect network effect values in the results of the traditional pairwise meta-analysis and the network meta-analysis (see Supplementary Table 1). Specifically, no inconsistency was found in either efficacy (*P* = 0.054) or safety (*P* = 0.819) within Chi-square tests. The loop-specific approach did not present any statistically significant inconsistency.

#### Ranking

**Figure 4:**
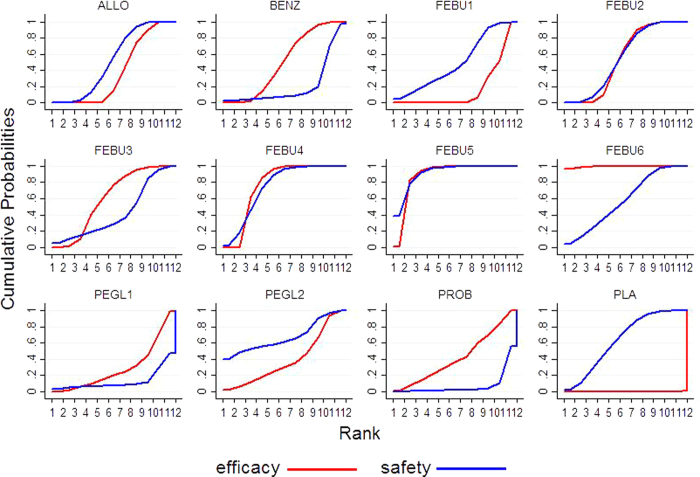
Cumulative efficacy and safety rankings of urate-lowing drugs. ALLO = allopurinol, FEBU1 = febuxostat 20 mg/day, FEBU2 = febuxostat 40 mg/day, FEBU3 = febuxostat 60 mg/day, FEBU4 = febuxostat 80 mg/day, FEBU5 = febuxostat 120 mg/day, FEBU6 = febuxostat 240 mg/day, BENZ = benzbromarone, PROB = probenecid, PEGL1 = pegloticase 8 mg every 2 weeks, PEGL2 = pegloticase 8 mg every 4 weeks, PLA = placebo.

Cumulative ranking plots of each treatment for efficacy and safety are shown in Fig. 4. Febuxostat 80/120 mg QD provided excellent efficacy and safety with a large area under both curves. Details of the SUCRA percentages and the calculated ranks are available in Supplementary Table 2. In terms of efficacy, the SUCRAs for febuxostat 240/120/80/60/40 mg QD, benzbromarone, probenecid, pegloticase 8 mg 4 W, allopurinol, pegloticase 8 mg 2 W, febuxostat 20 mg QD and placebo were 99.5%, 88.7%, 76.7%, 61.6%, 55.0%, 50.5%, 42.4%, 39.7%, 38.6%, 29.7%, 17.5% and 0.1%, respectively. Regarding safety, the cumulative probabilities of the treatments were 91.5%, 74.9%, 67.5%, 64.2%, 57.8%, 56.1%, 52.8%, 50.0%, 42.9%, 21.6%, 13.0% and 7.8% for febuxostat 120/80 mg QD, pegloticase 8 mg 4 W, placebo, febuxostat 240/40 mg QD, allopurinol, febuxostat 20/60 mg QD, benzbromarone, pegloticase 8 mg 2 W and probenecid, respectively.

**Figure 5:**
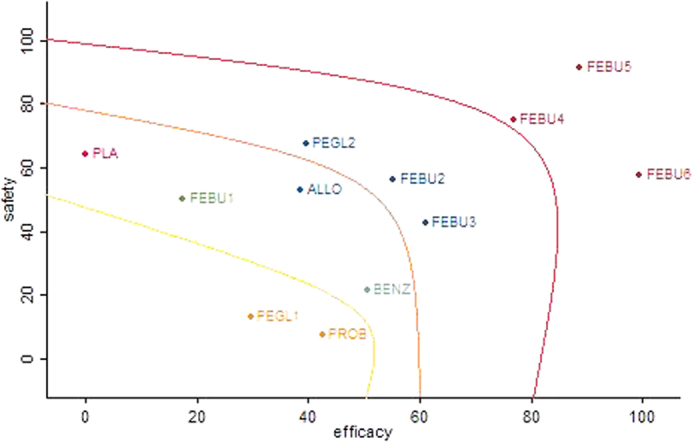
Clustered ranking plot for efficacy and safety of urate-lowing drugs. ALLO = allopurinol, FEBU1 = febuxostat 20 mg/day, FEBU2 = febuxostat 40 mg/day, FEBU3 = febuxostat 60 mg/day, FEBU4 = febuxostat 80 mg/day, FEBU5 = febuxostat 120 mg/day, FEBU6 = febuxostat 240 mg/day, BENZ = benzbromarone, PROB = probenecid, PEGL1 = pegloticase 8 mg every 2 weeks, PEGL2 = pegloticase 8 mg every 4 weeks, PLA = placebo.

Utilizing the SUCRA values, we displayed a clustered ranking plot of these treatments in the two dimensions of the x-axis (efficacy) and the y-axis (safety) in Fig. 5. Febuxostat was superior to the other drugs in both efficacy and safety, especially febuxostat 120 mg QD. Allopurinol took a medium position in the benefits and harms ranking. Compared with pegloticase 8 mg 2 W, pegloticase 8 mg 4 W showed better efficacy and safety. Benzbromarone and probenecid were likely to have similar rankings with an overall moderate benefit. However, probenecid ranked the worst for safety.

## Discussion

Using a network meta-analysis approach, we found that febuxostat tended to have higher efficacy and safety than other urate-lowering drugs, especially at a dose of 120 mg once daily. There was no evidence suggesting that adverse drug events outweighed the benefits of any of the five categories of ULT other than probenecid.

Probenecid was introduced as a uricosuric drug in 1951, and it is generally applicable for patients who cannot tolerate XOIs or fail to achieve their target serum urate acid with them^15^,^16^. Allopurinol, a purine analogue, has been widely used as a hypouricemic drug since the 1960s and was approved by the US Food and Drug Administration (FDA) in 1965. However, patients taking allopurinol have a high risk of serious hypersensitivity syndromes, such as Stevens-Johnson syndrome and toxic epidermal necrolysis, which may have a strong association with the HLA-B*5801 allele, a genetic change more commonly observed in Asian populations^19^,^54^. Benzbromarone was introduced as a uricosuric drug in the 1970s. It was widely registered in countries throughout Europe, Asia and South America before being withdrawn from the European market in 2003 due to its serious hepatotoxicity^17^. Febuxostat, a non-purine selective inhibitor of xanthine oxidase, has been approved by the European Medicines Agency (EMA) since 2008 and by the US-FDA since 2009. Considering that febuxostat is far more expensive than allopurinol^15^, it is often used when allopurinol is contraindicated or not tolerated^55^. Pegloticase, a new anti-hyperuricemia drug, was introduced to markets in 2010 by the FDA, and only one report including two placebo-controlled RCTs was reported in 2011^45^. To be noted, immunogenic responses to pegloticase should be monitored because it is a recombinant porcine-like uricase.

Several meta-analyses of RCTs have attempted to address comparative effects of urate-lowering drugs. A Cochrane systematic review compared febuxostat against allopurinol in achievement of urate-lowering target levels (relative risk (RR) of febuxostat 80 mg *vs.* allopurinol: 1.5, 95% CI: 1.2–1.8) and (RR of febuxostat 120 mg *vs.* allopurinol: 2.6, 95% CI: 2.0–3.3), which outcome was measured by an opposite indicator from ours. Regarding the occurrence of adverse events, there was a lower rate when comparing febuxostat 80 mg and 120 mg against allopurinol (RR: 0.93, 95% CI: 0.87–0.99, and RR: 0.90, 95% CI: 0.84–0.96, respectively)^56^. Additionally, a previous meta-analysis including five trials compared febuxostat with allopurinol in urate-lowering efficacy (RR: 1.56, 95% CI: 1.22–2.00, its efficacy outcome by the proportion of patients meeting the therapeutic target for serum uric acid level) and risk of adverse events (RR: 0.94, 95% CI: 0.90–0.99)^24^. Using a more advanced approach, our study found that febuxostat had an advantage over allopurinol in urate-lowering efficacy and safety. According to the guidelines of ACR and the European League against Rheumatism (EULAR), XOIs such as allopurinol and febuxostat are recommended for use prior to uricosuric agents and uricase for ULT^16^,^57^. Therefore, it is also worthwhile to discuss the efficacy and safety of uricosuric agents and uricase.

According to our ranking of efficacy, benzbromarone was only second to febuxostat at achieving urate-lowering targets. Benzbromarone has performed excellently at promoting the excretion of uric acid despite life-threatening adverse events reported in the past^17^. Essential guidelines have been recommended to prevent benzbromarone hepatotoxicity such as regularly monitoring liver function^57^. Limited clinical trials have been carried out with benzbromarone and probenecid, partially owing to the impact of being withdrawn from the market, the development of new drugs, regional/ethnic differences, prescribing habits and cost. Uricase-based drugs can metabolize uric acid to allantoin, which reduces the risk of precipitate. In addition, short-term trials have shown their urate-lowering effectiveness. However, our study did not reveal any significant differences in comparisons of pegloticase against other drugs.

Our study has clinical implications. The prevalence of gout and hyperuricemia has increased around both developed and developing countries, presumably due to lifestyle changes^3^,^10^. Hyperuricemia is associated with metabolic syndromes such as hypertension, dyslipidaemia, obesity and diabetes^10^,^11^,^12^ and with renal and cardiovascular diseases^13^,^14^,^15^. More and more patients need urate-lowering treatment. It is essential to know the comparative effects and safety of urate-lowering drugs available in the market. Our study pooled and ranked the efficacy and safety of these drugs using the data from individual RCTs, and thus our findings may be useful to clinicians in their decisions on which drug to use.

Our study has strengths. We designed our network meta-analysis as standardized by the PRISMA principle and conducted it carefully to minimize errors and ensure the validity of findings from all relevant studies identified. To our knowledge, our network meta-analysis is the first to address comparative effects of different ULTs with explicit rankings of efficacy and safety of different ULTs. We look forward to using this network-based statistical method to combine findings from individual studies and provide useful information for clinical decision-making. Finally, we analysed all of the trials of ULTs being used commonly, and we came to the conclusion that febuxostat had better urate-lowering effects than other drugs.

There are some limitations to our study. Firstly, this study included a limited number of trials. On the one hand, some drugs were only used in limited countries and areas, e.g., benzbromarone. On the other hand, we set language restrictions and excluded studies not in English. Secondly, some estimated results of the network meta-analysis relied on indirect comparisons. However, our results from direct comparisons were in accordance with the indirect and mixed comparisons. No obvious evidence suggesting inconsistency was found by fitting the inconsistency model. Thirdly, medicines with specific indications and some new drugs under development were not considered. With the improvement and application of network-based approaches, we promise to implement further predictions for drug/genome-target interactions with known reachable paths in the network and provide better interpretations for decision-makers.

## Conclusions

In conclusion, this systematic review and network meta-analysis provides clear evidence of the efficacy and safety of ULT. When comparing the ability to achieve sUA treatment targets and the occurrence of adverse events, febuxostat ranked first among the urate-lowering drugs. Benzbromarone and probenecid had moderate therapeutic effects, but they caused unpleasant side effects. Comprehensively considered, our findings support the recommendation of XOIs such as febuxostat and allopurinol. Pegloticase and similar new uricase drugs need further investigation through RCTs and meta-analyses.

## Additional Information

**How to cite this article**: Li, S. *et al*. Comparative efficacy and safety of urate-lowering therapy for the treatment of hyperuricemia: a systematic review and network meta-analysis. *Sci. Rep.* **6**, 33082; doi: 10.1038/srep33082 (2016).

## Supplementary Material

Supplementary Information

## Figures and Tables

**Table 1:** Summary of randomized controlled trials.

NO.	Study ID	Year	Duration of trials (weeks)	Location	No. of patients	N	Intervention	Drug dosing	Age 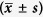	Male n(%)	sUA baseline (mg/dl)
1	Becker 2005a	2005	52	US and Canada	762	257	febuxostat	80 mg/day	51.8 ± 11.7	243(95)	9.80 ± 1.24
						251	febuxostat	120 mg/day	52.0 ± 12.1	243(97)	9.84 ± 1.26
						254	allopurinol	300 mg/day	51.6 ± 12.6	243(96)	9.90 ± 1.23
2	Becker 2005b	2005	4	US	153	37	febuxostat	40 mg/day	52.2 ± 14.0	33(89)	9.24 ± 1.33
						40	febuxostat	80 mg/day	55.2 ± 13.1	38(95)	9.92 ± 1.30
						38	febuxostat	120 mg/day	56.2 ± 10.8	33(87)	9.58 ± 1.11
						38	placebo	—	52.4 ± 12.6	32(84)	9.87 ± 1.33
3	Becker 2010	2010	26	US	2268	757	febuxostat	40 mg/day	32.9 ± 6.4	722(95)	9.60 ± 1.15
						756	febuxostat	80 mg/day	32.9 ± 6.4	710(94)	9.60 ± 1.20
						755	allopurinol	300/200 mg/day	32.7 ± 6.2	709(94)	9.50 ± 1.19
4	Huang 2014	2014	24/12	China	516	172	febuxostat	40 mg/day	46.4 ± 10.9	167(97)	9.89 ± 1.36
						172	febuxostat	80 mg/day	47.4 ± 11.2	169(98)	9.98 ± 1.39
						172	allopurinol	300 mg/day	46.2 ± 11.6	168(98)	9.95 ± 1.35
5	Kamatani 2011a	2011	16	Japan	40	10	febuxostat	40 mg/day	56.0 ± 8.2	10(100)	8.64 ± 0.77
						10	febuxostat	60 mg/day	53.3 ± 11.0	9(90)	8.48 ± 1.15
						20	allopurinol	300 mg/day	51.3 ± 12.0	19(95)	8.34 ± 1.16
6	Kamatani 2011b	2011	8	Japan	244	122	febuxostat	40 mg/day	51.6 ± 13.1	119(98)	8.94 ± 1.06
						122	allopurinol	200 mg/day	52.6 ± 14.0	118(97)	8.92 ± 0.87
7	Kamatani 2011c	2011	16	Japan	202	43	febuxostat	20 mg/day	52.1 ± 14.0	41(95)	8.80 ± 1.01
						41	febuxostat	40 mg/day	54.0 ± 11.8	41(100)	8.85 ± 0.89
						38	febuxostat	60 mg/day	51.2 ± 11.9	35(92)	8.76 ± 0.98
						42	febuxostat	80 mg/day	49.9 ± 12.8	40(95)	8.76 ± 1.05
						38	placebo	—	56.1 ± 13.3	37(97)	8.84 ± 1.02
8	Kamatani 2011d	2011	8	Japan	102	35	febuxostat	20 mg/day	50.9 ± 14.0	35(100)	8.83 ± 0.63
						34	febuxostat	40 mg/day	43.3 ± 13.6	34(100)	8.84 ± 0.82
						33	placebo	—	48.2 ± 13.4	33(100)	8.95 ± 0.99
9	Perez-Ruiz 1999	1999	36	Europe	36	19	allopurinol	100–300 mg/day	67.3 ± 9.59	NA	8.96 ± 1.84
						17	benzbromarone	100–200 mg/day	60.9 ± 12.8	NA	9.35 ± 1.96
10	Reinders 2009a	2009	8	Europe	62	27	benzbromarone	200 mg/day	55.0 ± 16.0	27(100)	9.17 ± 1.50
						35	probenecid	2 g/day	58.0 ± 12.0	33(94)	9.00 ± 1.17
11	Reinders 2009b	2009	16–8	Europe	65	36	allopurinol	300–600 mg/day	58.6 ± 12.3	29(81)	9.00 ± 1.50
						29	benzbromarone	100–200 mg/day	59.6 ± 11.3	24(83)	8.50 ± 1.33
12	Schumacher 2008	2008	28	US	1072	267	febuxostat	80 mg/day	51.0 ± 12.0	251(94)	9.85 ± 1.26
						269	febuxostat	120 mg/day	51.0 ± 12.0	255(95)	
						134	febuxostat	240 mg/day	54.0 ± 13.0	126(94)	
						268	allopurinol	300 mg/day	52.0 ± 12.0	249(93)	
						134	placebo	—	52.0 ± 12.0	123(92)	
13	Sundy 2011	2011	24	US, Canada, and Mexico	225	90	pegloticase	8 mg every 2 weeks	56.3 ± 15.5	68(80)	9.65 ± 1.65
						89	pegloticase	8 mg every 4 weeks	54.5 ± 13.3	69(82)	9.99 ± 1.78
						46	placebo	—	55.4 ± 12.2	36(84)	9.61 ± 1.59
14	Xu 2015	2015	24	China	504	168	febuxostat	40 mg/day	45.5 ± 11.9	158(99)	9.35 ± 1.22
						168	febuxostat	80 mg/day	48.2 ± 12.0	146(92)	9.42 ± 1.26
						168	allopurinol	300 mg/day	46.6 ± 10.7	149(94)	9.57 ± 1.30
15	Yu2016	2016	12	Taiwan, China	109	54	febuxostat	80 mg/day	46.0 ± 11.0	53(98.1)	83.3%£
						55	allopurinol	300 mg/day	45.2 ± 12.0	53(96.4)	80.0%£

NA = not available.

^£^The proportion of subjects with serum urate levels ≥9 mg/dL at baseline was 83.3% in the febuxostat group and 80.0% in the allopurinol group.

**Table 2:** Efficacy and safety of different drugs according to pairwise estimates.

Intervention	Pairwise meta-analysis odds ratio (and 95% CI)	No. of participants	No. of trials	No. of events	Heterogeneity I^2^ (variation in OR attributable to heterogeneity)
**Efficacy**					
allopurinol *vs.*					
Benzbromarone	2.28 (0.21, 24.64)	92	2	29	73.7%
febuxostat 40 mg QD	**1.29 (1.05, 1.59)**	2442	5	1364	14.2%
febuxostat 60 mg QD	8.13 (0.39, 167.90)	24	1	5	NA
febuxostat 80 mg QD	**3.62 (2.69, 4.89)**	3287	6	100	69.3%
febuxostat 120 mg QD	**6.34 (4.79, 8.40)**	1012	2	420	0.0%
febuxostat 240 mg QD	**18.31 (9.17, 36.58)**	389	1	171	NA
Placebo	**0.01 (0.00, 0.09)**	390	1	287	NA
benzbromarone *vs.*					
Probenecid	0.63 (0.05, 7.39)	55	1	3	NA
febuxostat 20 mg QD *vs.*					
febuxostat 40 mg QD	**7.39 (3.29, 16.63)**	153	2	52	0.0%
febuxostat 60 mg QD	**5.75 (1.99, 16.63)**	79	1	29	NA
febuxostat 80 mg QD	**8.28 (2.73, 25.15)**	84	1	28	NA
Placebo	**0.03 (0.00, 0.14)**	149	2	112	0.0%
febuxostat 40 mg QD *vs.*					
febuxostat 60 mg QD	1.16 (0.38, 3.58)	94	2	14	0.0%
febuxostat 80 mg QD	**2.28 (1.92, 2.70)**	2328	5	1073	0.0%
febuxostat 120 mg QD	**12.63 (2.60, 61.38)**	68	1	17	NA
Placebo	**0.00 (0.00, 0.02)**	215	3	130	0.0%
febuxostat 60 mg QD *vs.*					
febuxostat 80 mg QD	1.44 (0.40, 5.19)	77	1	11	NA
Placebo	**0.01 (0.00, 0.05)**	74	1	43	NA
febuxostat 80 mg QD *vs.*					
febuxostat 120 mg QD	**1.48 (1.05, 2.08)**	1080	3	250	18.6%
febuxostat 240 mg QD	**4.44 (2.20, 8.96)**	379	1	80	NA
Placebo	**0.00 (0.00, 0.01)**	531	3	282	0.0%
febuxostat 120 mg QD *vs.*					
febuxostat 240 mg QD	**3.11 (1.53, 6.32)**	371	1	66	NA
Placebo	**0.00 (0.00, 0.01)**	461	2	219	0.0%
febuxostat 240 mg QD *vs.*					
Placebo	**0.00 (0.00, 0.01)**	253	1	136	NA
pegloticase 8 mg 2 W *vs.*					
pegloticase 8 mg 4 W	1.39 (0.75, 2.60)	169	1	104	NA
Placebo	**0.02 (0.00, 0.36)**	127	1	98	NA
pegloticase 8 mg 4 W *vs.*					
Placebo	**0.02 (0.00, 0.26)**	128	1	92	NA
**Safety**					
allopurinol *vs.*					
Benzbromarone	0.29 (0.05, 1.62)	55	1	7	NA
febuxostat 40 mg QD	0.99 (0.84, 1.16)	2436	4	1345	0.0%
febuxostat 80 mg QD	1.17 (0.99, 1.38)	3345	6	2024	12.3%
febuxostat 120 mg QD	**1.56 (1.17, 2.08)**	1040	2	787	0.0%
febuxostat 240 mg QD	1.08 (0.67, 1.73)	402	1	298	NA
Placebo	1.12 (0.70, 1.79)	402	1	297	NA
benzbromarone *vs.*					
Probenecid	0.42 (0.12, 1.41)	55	1	17	NA
febuxostat 20 mg QD *vs.*					
febuxostat 40 mg QD	1.27 (0.64, 2.51)	153	2	102	0.0%
febuxostat 60 mg QD	0.84 (0.33, 2.14)	79	1	51	NA
febuxostat 80 mg QD	1.08 (0.45, 2.61)	84	1	52	NA
Placebo	1.10 (0.54, 2.22)	149	2	102	0.0%
febuxostat 40 mg QD *vs.*					
febuxostat 60 mg QD	0.78 (0.31, 1.99)	77	1	49	NA
febuxostat 80 mg QD	1.09 (0.93, 1.29)	2352	5	1241	0.0%
febuxostat 120 mg QD	1.18 (0.48, 2.91)	75	1	39	NA
Placebo	0.96 (0.56, 1.68)	221	3	135	0.0%
febuxostat 60 mg QD *vs.*					
febuxostat 80 mg QD	1.28 (0.50, 3.26)	77	1	49	NA
placebo	1.45 (0.56, 3.75)	74	1	46	NA
febuxostat 80 mg QD *vs.*					
febuxostat 120 mg QD	1.14 (0.87, 1.48)	1121	3	800	0.0%
febuxostat 240 mg QD	0.77 (0.49, 1.22)	401	1	279	NA
placebo	0.93 (0.64, 1.35)	558	3	367	0.0%
febuxostat 120 mg QD *vs.*					
febuxostat 240 mg QD	0.78 (0.49, 1.24)	403	1	281	NA
placebo	0.85 (0.56, 1.27)	479	3	318	0.0%
febuxostat 240 mg QD *vs.*					
placebo	1.04 (0.61, 1.78)	268	1	195	NA
pegloticase 8 mg 2 W *vs.*					
pegloticase 8 mg 4 W	11.55 (0.63, 212.19)	169	1	164	NA
placebo	10.18 (0.48, 216.91)	127	1	125	NA
pegloticase 8 mg 4 W *vs.*					
placebo	0.78 (0.15, 4.20)	128	1	121	NA

NA = not available.
