# Supplementary material for: Comparative efficacy and safety of urate-lowering therapy for the treatment of hyperuricemia: a systematic review and network meta-analysis
Source: Sci Rep. 2016 Sep 8;6:33082. doi: 10.1038/srep33082 (PMC5015109; doi:10.1038/srep33082)
Supplement: Supplementary Information [file srep33082-s1.pdf]

Comparative efficacy and safety of urate-lowering therapy for the treatment of hyperuricemia: a systematic review and network meta-analysis

Shu Li[1,2*], Hongxi Yang[1,2*], Fengjiang Wei[3], Xilin Yang[4], Daiqing Li[2], Mingzhen Li[2], Weili Xu[4,5], Weidong Li[3], Li Sun[1], Yanan Guo[1], Ying Gao[1] & Yaogang Wang[1+]

Appendix

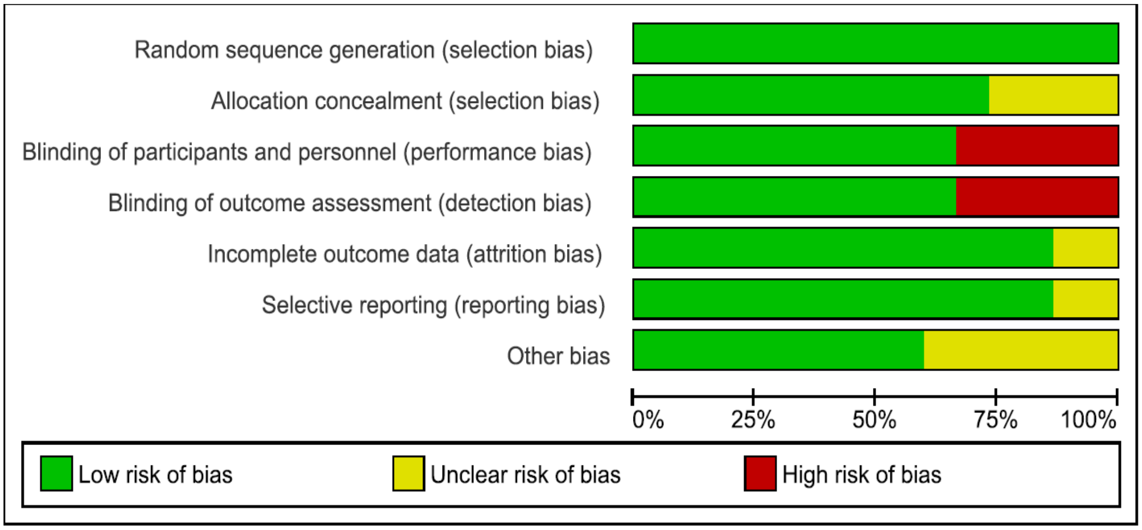

Supplementary Figure 1A. Risk of bias graph of included studies on overall level.

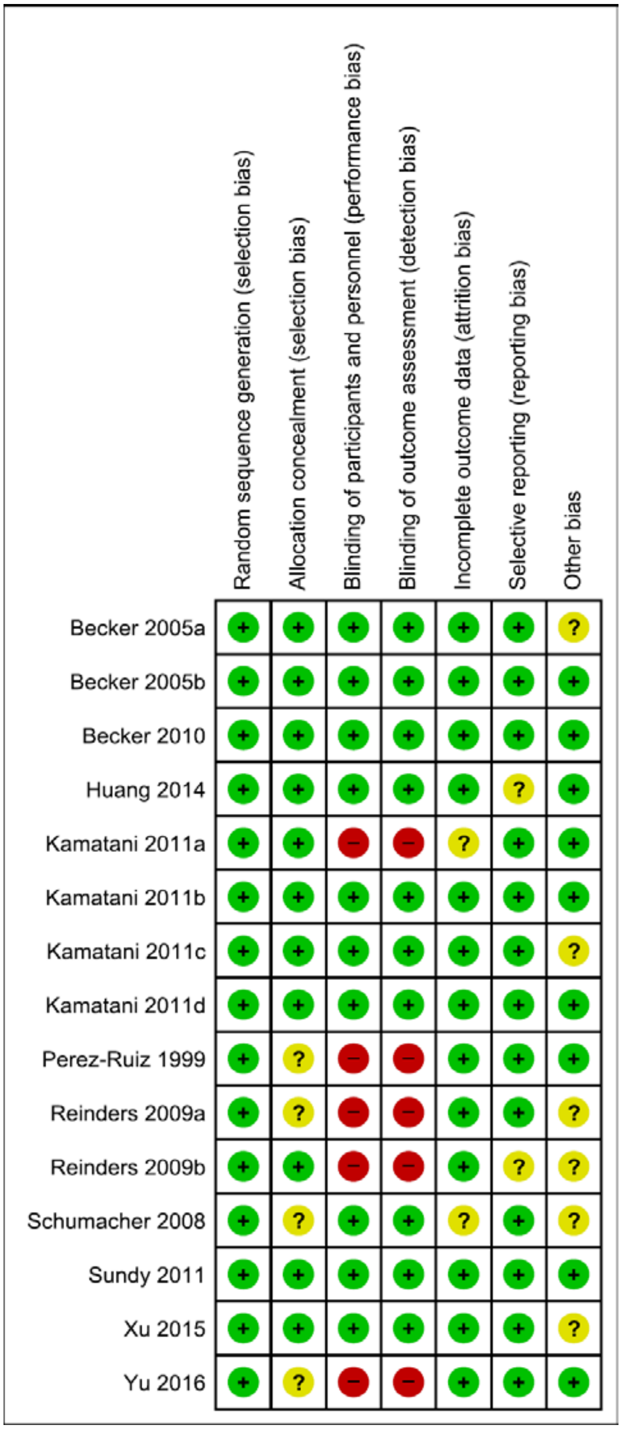

Supplementary Figure 1B. Risk of bias summary of included studies.

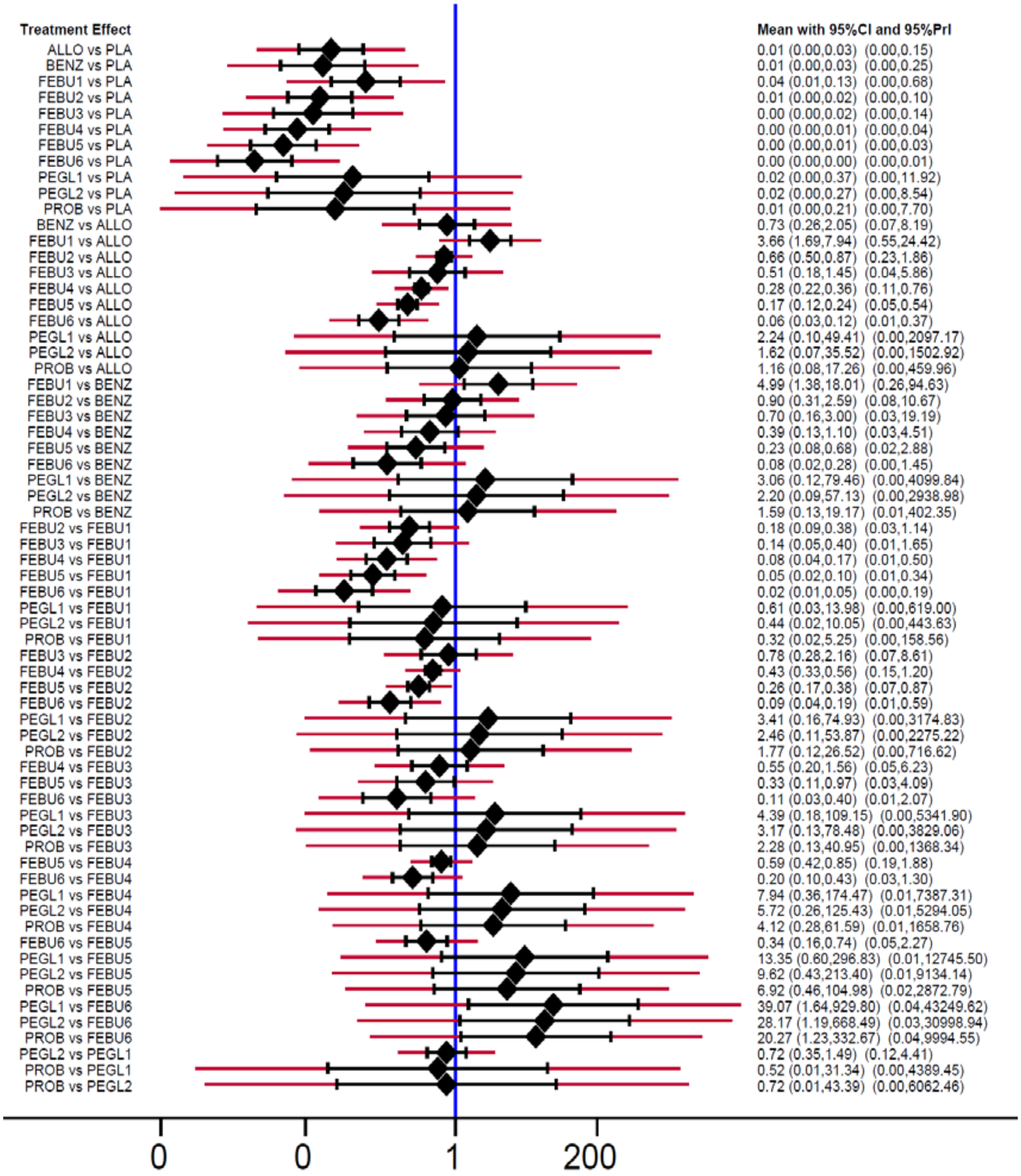

Supplementary Figure 2. Predictive interval plot for urate-lowering drugs on a logarithmic scale. (ALLO=allopurinol, FEBU1=febuxostat 20 mg/day, FEBU2=febuxostat 40 mg/day, FEBU3=febuxostat 60 mg/day, FEBU4=febuxostat 80 mg/day, FEBU5=febuxostat 120 mg/day, FEBU6=febuxostat 240 mg/day, BENZ=benzbromarone, PROB=probenecid, PEGL1=pegloticase 8 mg every 2 weeks, PEGL2=pegloticase 8 mg every 4 weeks, PLA=placebo.)

Intervention	Direct comparisons/ participants (n/N)	Odds ratio (95% CI)	
		Pairwise meta-analysis	Network meta-analysis
**Efficacy**			
allopurinol *vs.*			
benzbromarone	29/92	2.28 (0.21, 24.64)	1.36 (0.49,3.81)
febuxostat 20 mg QD	—	—	0.27 (0.13,0.59)
febuxostat 40 mg QD	1364/2442	1.29 (1.05, 1.59)	1.52 (1.15,1.99)
febuxostat 60 mg QD	5/24	8.13 (0.39, 167.90)	2.01 (0.71,5.69)
febuxostat 80 mg QD	100/3287	3.62 (2.69, 4.89)	3.54 (2.80,4.47)
febuxostat 120 mg QD	420/1012	6.34 (4.79, 8.40)	5.95 (4.15,8.52)
febuxostat 240 mg QD	171/389	18.31 (9.17, 36.58)	17.41 (8.22,36.89)
pegloticase 8 mg 2W	—	—	0.45 (0.02,9.85)
pegloticase 8 mg 4W	—	—	0.62 (0.03,13.63)
probenecid	—	—	0.86 (0.06,12.72)
placebo	287/390	0.01 (0.00, 0.09)	0.01 (0.00,0.03)
benzbromarone *vs.*			
febuxostat 20 mg QD	—	—	0.20 (0.06,0.73)
febuxostat 40 mg QD	—	—	1.11 (0.39,3.21)
febuxostat 60 mg QD	—	—	1.47 (0.34,6.35)
febuxostat 80 mg QD	—	—	2.60 (0.91,7.43)
febuxostat 120 mg QD	—	—	4.37 (1.47,12.93)
febuxostat 240 mg QD	—	—	12.78 (3.58,45.60)
pegloticase 8 mg 2W	—	—	0.33 (0.01,8.54)
pegloticase 8 mg 4W	—	—	0.46 (0.02,11.81)
probenecid	3/55	0.63 (0.05, 7.39)	0.63 (0.05,7.62)
placebo	—	—	0.01 (0.00,0.03)
febuxostat 20 mg QD *vs.*			
febuxostat 40 mg QD	52/153	7.39 (3.29, 16.63)	5.52 (2.62,11.64)
febuxostat 60 mg QD	29/79	5.75 (1.99, 16.63)	7.32 (2.54,21.06)
febuxostat 80 mg QD	28/84	8.28 (2.73, 25.15)	12.88 (6.01,27.60)
febuxostat 120 mg QD	—	—	21.66 (9.49,49.42)
febuxostat 240 mg QD	—	—	63.40 (22.00,182.68)
pegloticase 8 mg 2W	—	—	1.63 (0.07,37.18)
pegloticase 8 mg 4W	—	—	2.26 (0.10,51.45)
probenecid	—	—	3.13 (0.19,51.56)
placebo	112/149	0.03 (0.00, 0.14)	0.04 (0.01,0.13)
febuxostat 40 mg QD *vs.*			
febuxostat 60 mg QD	14/94	1.16 (0.38, 3.58)	1.32 (0.48,3.69)
febuxostat 80 mg QD	1073/2328	2.28 (1.92, 2.70)	2.33 (1.79,3.03)
febuxostat 120 mg QD	17/68	12.63 (2.60, 61.38)	3.92 (2.62,5.88)
febuxostat 240 mg QD	—	—	11.48 (5.27,25.01)
pegloticase 8 mg 2W	—	—	0.30 (0.01,6.49)
pegloticase 8 mg 4W	—	—	0.41 (0.02,8.97)

probenecid	—	—	0.57 (0.04,8.49)
placebo	130/215	0.00 (0.00, 0.02)	0.01 (0.00,0.02)
febuxostat 60 mg QD *vs.*			
febuxostat 80 mg QD	11/77	1.44 (0.40, 5.19)	1.76 (0.63,4.94)
febuxostat 120 mg QD	—	—	2.96 (1.01,8.72)
febuxostat 240 mg QD	—	—	8.67 (2.44,30.78)
pegloticase 8 mg 2W	—	—	0.22 (0.01,5.53)
pegloticase 8 mg 4W	—	—	0.31 (0.01,7.66)
probenecid	—	—	0.43 (0.02,7.67)
placebo	43/74	0.01 (0.00, 0.05)	0.00 (0.00,0.02)
febuxostat 80 mg QD *vs.*			
febuxostat 120 mg QD	250/1080	1.48 (1.05, 2.08)	1.68 (1.18,2.40)
febuxostat 240 mg QD	80/379	4.44 (2.20, 8.96)	4.92 (2.32,10.43)
pegloticase 8 mg 2W	—	—	0.13 (0.01,2.78)
pegloticase 8 mg 4W	—	—	0.18 (0.01,3.85)
probenecid	—	—	0.24 (0.02,3.63)
placebo	282/531	0.00 (0.00, 0.01)	0.00 (0.00,0.01)
febuxostat 120 mg QD *vs.*			
febuxostat 240 mg QD	66/371	3.11 (1.53, 6.32)	2.93 (1.35,6.33)
pegloticase 8 mg 2W	—	—	0.08 (0.00,1.67)
pegloticase 8 mg 4W	—	—	0.10 (0.00,2.31)
probenecid	—	—	0.14 (0.01,2.19)
placebo	219/461	0.00 (0.00, 0.01)	0.00 (0.00,0.01)
febuxostat 240 mg QD *vs.*			
pegloticase 8 mg 2W	—	—	0.03 (0.00,0.61)
pegloticase 8 mg 4W	—	—	0.04 (0.00,0.85)
probenecid	—	—	0.05 (0.00,0.81)
placebo	136/253	0.00 (0.00, 0.01)	0.00 (0.00,0.00)
pegloticase 8 mg 2W *vs.*			
pegloticase 8 mg 4W	104/169	1.39 (0.75, 2.60)	1.39 (0.67,2.87)
probenecid	—	—	1.92 (0.03,115.82)
placebo	98/127	0.02 (0.00, 0.36)	0.02 (0.00,0.37)
pegloticase 8 mg 4W *vs.*			
probenecid	—	—	1.38 (0.02,83.32)
placebo	92/128	0.02 (0.00, 0.26)	0.02 (0.00,0.27)
probenecid *vs.*			
placebo	—	—	0.01 (0.00,0.22)
**Safety**			
allopurinol *vs.*			
benzbromarone	7/55	0.29 (0.05, 1.62)	0.29 (0.05,1.62)
febuxostat 20 mg QD	—	—	0.91 (0.50,1.65)
febuxostat 40 mg QD	1345/2436	0.99 (0.84, 1.16)	1.02 (0.87,1.19)
febuxostat 60 mg QD	—	—	0.80 (0.36,1.76)
febuxostat 80 mg QD	2024/3345	1.17 (0.99, 1.38)	1.15 (0.99,1.32)
febuxostat 120 mg QD	787/1040	1.56 (1.17, 2.08)	1.39 (1.10,1.78)
febuxostat 240 mg QD	298/402	1.08 (0.67, 1.73)	1.02 (0.66,1.55)

pegloticase 8 mg 2W	—	—	0.11 (0.00,2.30)
pegloticase 8 mg 4W	—	—	1.22 (0.25,5.92)
probenecid	—	—	0.12 (0.01,1.00)
placebo	297/402	1.12 (0.70, 1.79)	1.08 (0.78,1.50)
benzbromarone *vs.*			
febuxostat 20 mg QD	—	—	3.18 (0.51,19.98)
febuxostat 40 mg QD	—	—	3.57 (0.62,20.41)
febuxostat 60 mg QD	—	—	2.79 (0.41,18.85)
febuxostat 80 mg QD	—	—	4.01 (0.70,22.92)
febuxostat 120 mg QD	—	—	4.88 (0.84,28.20)
febuxostat 240 mg QD	—	—	3.55 (0.59,21.24)
pegloticase 8 mg 2W	—	—	0.37 (0.01,12.69)
Pegloticase 8 mg 4W	—	—	4.28 (0.41,44.68)
probenecid	17/55	0.42 (0.12, 1.41)	0.42 (0.12,1.41)
placebo	—	—	3.77 (0.64,22.11)
febuxostat 20 mg QD *vs.*			
febuxostat 40 mg QD	102/153	1.27 (0.64, 2.51)	1.12 (0.62,2.02)
febuxostat 60 mg QD	51/79	0.84 (0.33, 2.14)	0.88 (0.37,2.10)
febuxostat 80 mg QD	52/84	1.08 (0.45, 2.61)	1.26 (0.70,2.28)
febuxostat 120 mg QD	—	—	1.53 (0.82,2.86)
febuxostat 240 mg QD	—	—	1.12 (0.55,2.28)
pegloticase 8 mg 2W	—	—	0.12 (0.01,2.64)
Pegloticase 8 mg 4W	—	—	1.34 (0.26,7.06)
probenecid	—	—	0.13 (0.01,1.19)
placebo	102/149	1.10 (0.54, 2.22)	1.19 (0.64,2.18)
febuxostat 40 mg QD *vs.*			
febuxostat 60 mg QD	49/77	0.78 (0.31, 1.99)	0.78 (0.36,1.72)
febuxostat 80 mg QD	1241/2352	1.09 (0.93, 1.29)	1.12 (0.96,1.31)
febuxostat 120 mg QD	39/75	1.18 (0.48, 2.91)	1.37 (1.05,1.78)
febuxostat 240 mg QD	—	—	1.00 (0.64,1.54)
pegloticase 8 mg 2W	—	—	0.10 (0.00,2.25)
pegloticase 8 mg 4W	—	—	1.20 (0.25,5.81)
probenecid	—	—	0.12 (0.01,0.98)
placebo	135/221	0.96 (0.56, 1.68)	1.06 (0.76,1.48)
febuxostat 60 mg QD *vs.*			
febuxostat 80 mg QD	49/77	1.28 (0.50, 3.26)	1.43 (0.65,3.15)
febuxostat 120 mg QD	—	—	1.75 (0.78,3.93)
febuxostat 240 mg QD	—	—	1.27 (0.53,3.07)
pegloticase 8 mg 2W	—	—	0.13 (0.01,3.14)
pegloticase 8 mg 4W	—	—	1.53 (0.27,8.71)
probenecid	—	—	0.15 (0.02,1.44)
placebo	46/74	1.45 (0.56, 3.75)	1.35 (0.60,3.02)
febuxostat 80 mg QD *vs.*			
febuxostat 120 mg QD	800/1121	1.14 (0.87, 1.48)	1.22 (0.96,1.54)
febuxostat 240 mg QD	279/401	0.77 (0.49, 1.22)	0.89 (0.58,1.35)

pegloticase 8 mg 2W	—	—	0.09 (0.00,2.00)
Pegloticase 8 mg 4W	—	—	1.07 (0.22,5.15)
probenecid	—	—	0.10 (0.01,0.87)
placebo	367/558	0.93 (0.64, 1.35)	0.94 (0.68,1.30)
febuxostat 120 mg QD *vs.*			
febuxostat 240 mg QD	281/403	0.78 (0.49, 1.24)	0.73 (0.47,1.13)
pegloticase 8 mg 2W	—	—	0.08 (0.00,1.65)
pegloticase 8 mg 4W	—	—	0.88 (0.18,4.26)
probenecid	—	—	0.09 (0.01,0.72)
placebo	318/479	0.85 (0.56, 1.27)	0.77 (0.54,1.10)
febuxostat 240 mg QD *vs.*			
pegloticase 8 mg 2W	—	—	0.10 (0.00,2.31)
pegloticase 8 mg 4W	—	—	1.20 (0.24,6.07)
probenecid	—	—	0.12 (0.01,1.02)
placebo	195/268	1.04 (0.61, 1.78)	1.06 (0.65,1.73)
pegloticase 8 mg 2W *vs.*			
pegloticase 8 mg 4W	164/169	11.55 (0.63, 212.19)	11.55 (0.63,212.19)
probenecid	—	—	1.12 (0.03,47.26)
placebo	125/127	10.18 (0.48, 216.91)	10.18 (0.48,216.91)
pegloticase 8 mg 4W *vs.*			
probenecid	—	—	0.10 (0.01,1.37)
placebo	121/128	0.78 (0.15, 4.20)	0.88 (0.19,4.12)
probenecid *vs.*			
placebo	—	—	9.06 (1.06,77.67)

Supplementary Table 1. Pairwise and network estimates of the effects of different drugs.

Intervention	Efficacy		Safety	
	SUCRA	Rank	SUCRA	Rank
allopurinol	38.6	9	52.8	7
benzbromarone	50.5	6	21.6	10
febuxostat 20 mg QD	17.5	11	50.0	8
febuxostat 40 mg QD	55.0	5	56.1	6
febuxostat 60 mg QD	61.6	4	42.9	9
febuxostat 80 mg QD	76.7	3	74.9	2
febuxostat 120 mg QD	88.7	2	91.5	1
febuxostat 240 mg QD	99.5	1	57.8	5
pegloticase 8 mg 2W	29.7	10	13.0	11
pegloticase 8 mg 4W	39.7	8	67.5	3
probenecid	42.4	7	7.8	12
placebo	0.1	12	64.2	4

Supplementary Table 2. Ranking of drugs according to efficacy and safety.
